# Aggressive chondroblastic osteosarcoma in a dog: A case report

**DOI:** 10.30466/vrf.2019.100779.2402

**Published:** 2019-12-15

**Authors:** Mohammad Reza Esmaili Nejad, Rana Vafaei, Majid Masoudifard, Seyed Mahdi Nassiri, Atena Salimi

**Affiliations:** 1 *Department of Surgery and Radiology, Faculty of Veterinary Medicine, University of Tehran, Tehran, Iran;*; 2 *Department of Clinical Pathology, Faculty of Veterinary Medicine, University of Tehran, Tehran, Iran.*

**Keywords:** Bone, Chondroblastic osteosarcoma, CT-Scan, Dog, Radiography

## Abstract

Canine osteosarcoma (OS) or osteogenic sarcoma is an aggressive tumor of the skeletal system, associated with a rapid progression and guarded prognosis. The osteosarcomas, mostly arise from the appendicular skeleton while axial OS (osteosarcoma of flat bones) are less reported in the majority of large breeds. This report describes complete para-clinical investigations of an aggressive chondroblastic OS involving facial flat bones with highly metastatic characterization in a large mix breed stray dog. Radiographic and computed tomography findings demonstrated an amorphous and active new bone formation, associated with the severe lytic areas in the left maxillary, orbital and zygomatic bones. Also, lots of nodular densities were distributed in all lung lobes. The cytological examination of the mass revealed individualized oval to spindle-shaped pleomorphic mesenchymal cells exhibiting many criteria of malignancy such as marked anisocytosis, anisokaryosis, prominent and multiple nucleoli. The punctate cytoplasmic vacuoles were obvious and bi-nucleated cells were frequently observed. These cells were seen in the background of an eosinophilic matrix. Histopathologic evaluation of the mass exhibited areas of osseous differentiation within the mass containing bony spicules and wavy bone formation along with the vast areas of cartilaginous differentiations with chondroblasts in lacunar spaces. Ultimately, chondroblastic OS with severe lung metastasis was confirmed and the animal was euthanized.

## Introduction

Osteosarcoma (OS) is a malignant primary bone tumor defined as a bone matrix-producing neoplasm arising from primitive transformed mesenchymal cells that differentiate into osteoblasts.^1^ In addition to producing an osteoid matrix, this tumor can also raise fibroblastic or cartilaginous matrix, therefore it has been divided into six subtypes of poorly differentiated, osteoblastic, chondroblastic, fibro-blastic, telangiectatic and giant cell forms.^2^ Combined type is referred to a subtype of OS which none of these patterns are dominant. Fibroblastic OS is found to have a more favorable prognosis than the other subtypes in dogs.^2^ Differently, chondroblastic OS has a high rate of metastasis. Metastasis to the lungs is more common, but it can occur to the other bones and organs as well.^3^ The lesion is not generally uniform and emerges as a severe, active and aggressive new bone formations and periosteal reactions. It has been rarely reported from domestic animals, but its characterizations are the same as in humans.^4^ The most predisposed breeds of dogs are large breeds and the mean age of the involvement is eight years.^5^ Axial OS is locally aggressive and destroys bone locally, it can invade into surrounding tissues and causes dystrophic calcification.^3^ Despite advances in various treatments (including amputation/sparing, chemotherapy and palliative radiotherapy) prognosis is still poor, with median survival time from three months to one year. This case report describes a metastatic form of chondroblastic OS, involving flat bones of the skull with severe lung metastasis in a young dog.

## Case Description

**Fig. 1:**
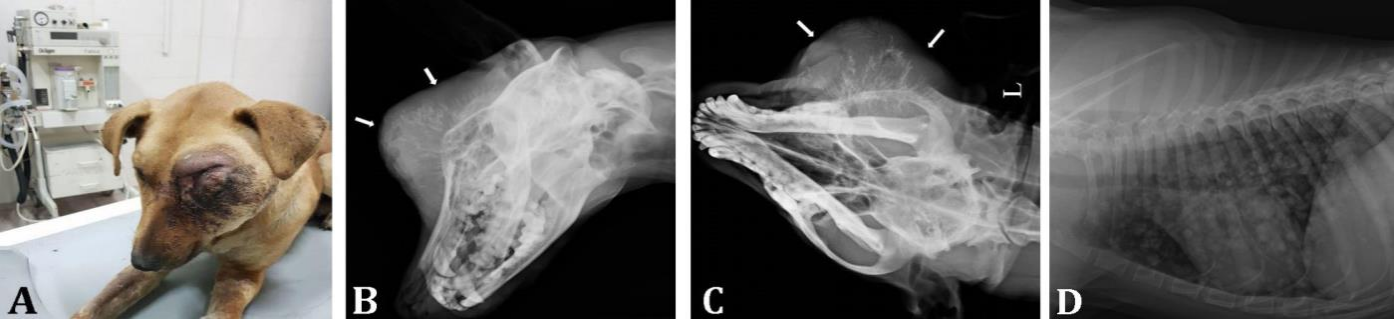
**A)** The gross image of the diseased dog. **B)** Lateral oblique and **C)** Dorso-ventral projection from the skull (aggressive destructive bony lesion, with a large amount of palisade and amorphous new bone formation, are noticeable around the white arrows). **D) **The left lateral radiograph demonstrating multiple cannon balls in lung.

A young (about 2-years-old) intact male, mixed-breed dog was referred to the Veterinary Teaching Hospital, University of Tehran with a history of weakness, lethargy, and depression, because of presence of a massive swelling with progressive growth around the left eye with subsequently deformity of the left orbital bone and loss of sight. Skin injuries due to the self-trauma in association with purulent secretions have been noticed during the clinical examination (Fig. 1A). The mass was painful and warm in clinical inspection at the time of presentation. Abnormal respiratory sounds were auscultated. Heart rate and rectal temperature were normal.

Standard digital radiographs using Direct-View, Classic CR System (Carestream Co., Montreal, Canada) were taken in lateral oblique and dorsoventral (DV) of the skull and left lateral, right lateral and ventrodorsal (VD) projections of the thoracic cavity (Kvp: 66.00, mAs: 3.20). Radiography of the skull revealed severe aggressive, amorphous and active new bone formations around the left zygomatic bone, orbital bone and maxillary sinus associated with regional soft tissue swelling (Figs. 1B and 1C). 

**Fig. 2:**
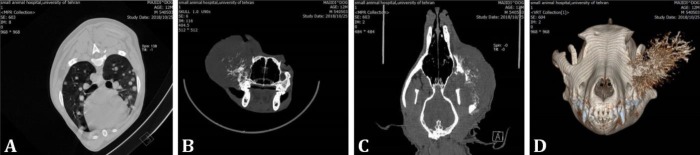
**A) **2-dimensional transverse plane CT-Scan from the thoracic cavity, a large number of the nodular interstitial pattern (cannon balls) are detectable in the lung lobes. The CT-Scan images of the skull including **B)** Transverse plane, **C)** Coronal plane and **D)** 3-dimensional reconstruction, severe active bony lysis, and irregular new bone formation in the left maxillary, orbital and zygomatic bones

Multiple cannon ball lesions with various size have also been noticed in the thoracic cavity in lung lobes. Heart shadow was normal in size (vertebral heart size: 10, 3^rd^ intercostal space) and shape. There was no sign of pleural effusion and pulmonary vessels, trachea, caudal vena cava and aortic diameter were normal subjectively (Figs. 1D and 2A). The CT-Scan procedures (plain and angiography) were performed to identify the exact location and extent of the lesion. To obtain CT-Scan images of the skull and thoracic cavity, the animal was positioned in dorsal recumbency. Anesthesia was performed using intravenous injection of ketamine (10.00 mg kg^-1^; Alfasan, Woerden, Netherlands) and acepromazine (0.10 mg kg^-1^; Alfasan). During anesthesia, continuous monitoring and observation were performed. The CT-Scan examination was performed using a multi-detectors helical machine (Somatom Spirit; Siemens, Munich, Germany) with the following setting: slice thickness: 1.00 mm; tube rotation time: 1 sec; pitch 1; 120.00 kVp; and 110 mAs. In addition, 750 mg kg^-1^ of iodinated contrast medium (Iohexol; GE Healthcare, Oklahoma city, USA) was injected intravenously to obtain post contrast images. After the imaging process, images were reconstructed to a high- spatial- frequency reconstruction algorithm (bone-U90) without additional filters. Furthermore, all images were reconstructed on a 2- dimensional (transverse, sagittal and coronal) and 3-dimensional viewing to allow a better understanding of the regional anatomy (Fig. 2). The CT-Scan has revealed amorphous periosteal reactions and new bone formation, long transitional zone with fuzzy margins indicating severely aggressive and active lesions, in the zygomatic, orbital, some parts of nasal and frontal bones. Soft tissue swelling and subsequently displacement of the left eye globe dorsally and medially were detectable as well. The left eye has been compressed by the mass and its normal structure has been altered. Increase regional attenuation following the contrast medium administration, indicated the high vascularization and the aggressiveness of the lesion. Also, the presence of discharge in the middle part of the left ear with thickening of tympanic bulla manifested chronic unilateral otitis media.

After CT-Scan examination and under general anesthesia, fine needle aspiration (FNA) from the mass was taken under ultrasound guidance. Doppler study during the ultrasound also showed the high vascularization of the lesion, although the deeper parts could not be evaluated due to far acoustic shadow produced by more superficial lesions. Multiple samples from different parts of the mass were sampled and sent to the laboratory for the cytological evaluations. Finally, the patient received high doses of anesthetic drugs for euthanasia upon the dog's owner requested. During necropsy, adequate amounts of the neoplastic and aggressive bone lesions were sampled for histopathologic examinations.

**Fig. 3:**
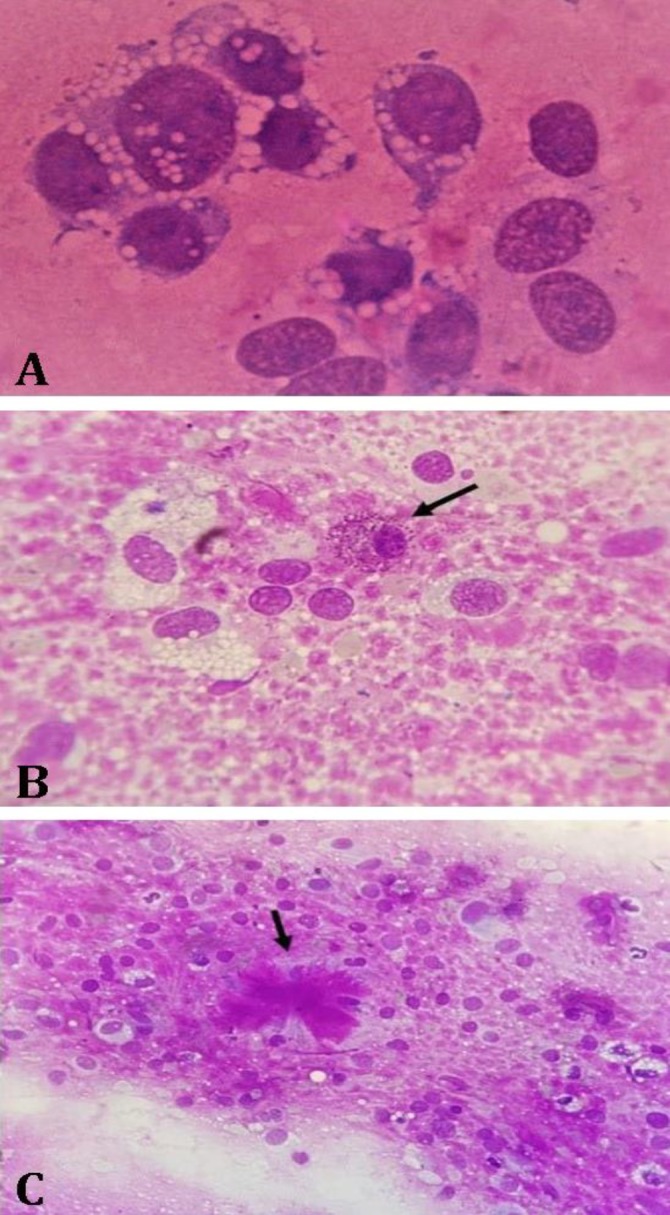
The cytologic characterizations of the tumor. **A)** close view of a group of individualized cells that are presumptively osteoblasts with a fine chondro-osseous material around them; **B)** The presence of a mast cell (arrow) among neoplastic cells; **C)** Osteoid matrix in a bizarre shape (arrow), (Giemsa, 1000×).

To evaluate the cytological features of the mass, FNA samples and impression smears from the tissue biopsy were prepared and stained with Giemsa. The stained tissue sections were highly cellular. Pleomorphic oval to spindle shaped mesenchymal cells exhibiting many criteria of malignancy such as marked anisocytosis, anisokaryosis, prominent and multiple nucleoli were observed. Punctate cytoplasmic vacuoles (Fig. 3A) and few mast cells were also present (Fig. 3B). The neoplastic cells were encompassed by their product; an abundant pink material in the shape of streams, swirls, pools and occasionally bizarre figures, resembling osteoid or chondroid matrix (Fig. 3C). 

**Fig. 4:**
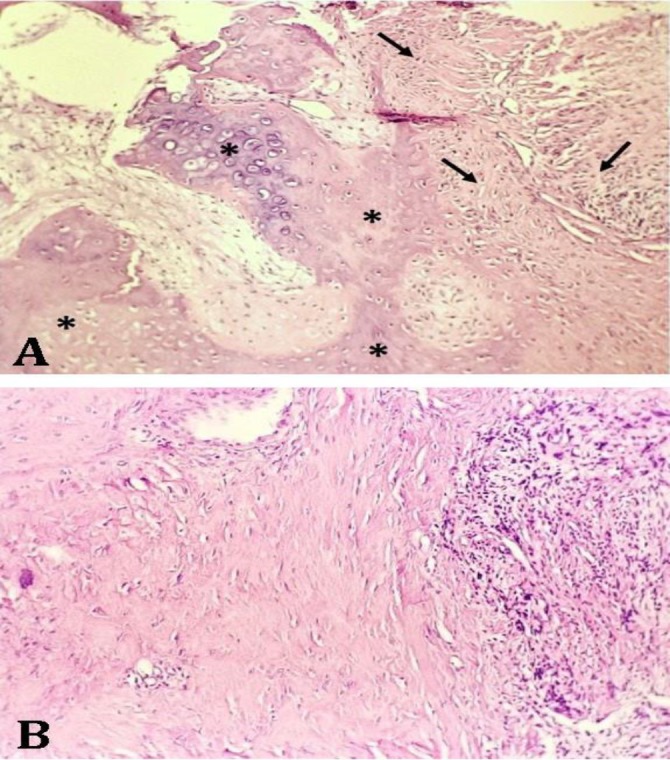
The histopathologic characteristics of the tumor. **A)** Different areas of tumor exhibiting a biphasic nature consisting of chondroblasts encompassed by the chondroid matrix (asterisks) and spindle-shaped neoplastic cells adjacent to the osteoid matrix (arrows); **B)** The areas of osseous formation in the left side of the figure and the pleomorphic neoplastic cells in the right side, (Hematoxylin and Eosin, 100×)

Binucleated cells were frequently seen in the tissue sections of the tumor. The nuclei had coarse nuclear chromatin containing multiple prominent nucleoli. The cytological findings confirmed the presence of a sarcoma most likely OS or chondrosarcoma. Histopathology examination showed a mesenchymal proliferation of neoplastic cells in different parts of the tumor. Areas of osseous differentiation within the mass containing bony spicules and wavy bone formation were associated with the vast areas of cartilaginous differentiation with chondroblasts in lacunar spaces (Fig. 4). Frequent mitotic figures were present in osseous parts.

## Discussion

Osteosarcoma is the most common neoplasm of all skeletal system tumors in dogs.^6^ About 90.00% of humans OSs are conventional OS and about 25.00% of them are chondroblastic OSs.^7^ Patnaik reviewed 14 cases of canine extra-skeletal OS and chondrosarcoma in which osteo-blastic OS were the most common type and there was just one case of chondroblastic OS that chondroid tissue was its predominant component.^8^ The FNA as a less invasive and less expensive examination^9^ was performed for this case owing to good sensitivity (86.00%) and specificity (95.00%) of cytology for diagnosis of OS, but the chondroid nature of the masses is challenging to clinical pathologists that requires histopathology examination.^7^^,^^10^ Although a tissue section from cartilaginous areas of the neoplastic mass would suggest an erroneous diagnosis of chondrosarcoma, which in such cases, radiographic findings such as aggressive appearance, lead to the diagnosis of chondroblastic OS.^11^ Generally, patients with axial skeleton tumor classified as cranial OS have a high rate of metastasis,^12^ which was observed in the present patient. In addition, the current patient notably showed clinical signs of lethargy, anorexia, weight loss and weakness in daily activities like the previously reported case of axial OS.^12^

Generally, the clinical presentation and various imaging modalities can lead to a presumptive diagnosis of OS, but the final diagnosis ultimately depends on histo-pathologic evaluation.^13^ Radiographically, OS presents a variation of lytic, mixed or productive patterns. However, lesions are not pathognomonic, and similar patterns can occur with other bone tumors. Further, radiographic appearance is dynamic and may change over time.^13^ Similarly, in the present patient, a mixed pattern of irregular lysis and amorphous new bone formation was detected in radio-graphy. Advanced imaging such as CT-Scan and magnetic resonance imaging have been demonstrated to play an important role in evaluating the extent of tumor for surgical planning; this has resulted in improved survival in OS of the mandible and maxilla in humans.^14^ The CT-Scan is the preferred imaging modality for biopsy planning in the maxilla-facial region. Furthermore, CT-Scan has increased sensitivity for the detection of pulmonary metastatic disease. In CT-Scan (plain and angiography) an aggressive bony proliferation and lysis, associated with a large number of cannon-ball with various sizes were notably visible in the current case. The type of tumor cannot be determined based on imaging diagnosis alone, and biopsy is always required for definitive diagnosis.^14^ It is important to consider the diagnostic imaging findings in light of clinical features including tumor location and the histopathological findings. The euthanasia was the owner's approach of choice because of the tumor extension into adjacent orbit regions, and also severe lung metastasis making the surgical procedure not viable.
